# Editorial: The Protein Alpha-Synuclein: Its Normal Role (in Neurons) and Its Role in Disease

**DOI:** 10.3389/fnins.2020.00116

**Published:** 2020-02-20

**Authors:** Ruth G. Perez

**Affiliations:** Department of Molecular and Translational Medicine, Graduate School of Biomedical Sciences, Paul L. Foster School of Medicine, Texas Tech University Health Sciences Center El Paso, El Paso, TX, United States

**Keywords:** alpha-synuclein function, alpha-synuclein aggregation, neurodegeneration, Parkinson's disease, synucleinopathies

The protein alpha-Synuclein (aSyn) is highly-studied due to its role in Parkinson's disease (PD) and its accrual as the major protein component of Lewy bodies/Lewy neurites (LB/LN) (Anderson et al., 2006). Though the precise role of aSyn in disease pathogenesis is not fully elucidated, aSyn toxicity is widely thought to be associated with its aggregation in LB/LN. This has led some to consider modulating aSyn expression as a means to counteract PD pathology. Yet, a multitude of studies show that altering aSyn expression or removing it from neurons has profound effects on many intracellular processes and in some cases induces neurodegeneration. This leads some to propose that normal aSyn function is crucial for particular neuronal populations and that PD results from a toxic loss of aSyn function (Perez and Hastings, 2004; Benskey et al., 2016). Indeed, aSyn is one of the most abundant proteins of the nervous system. Its role in neurotransmission at the synapse is well-established, and research also confirms roles for aSyn in neurotransmitter synthesis, calcium homeostasis, mitochondrial function, and gene regulation. Thus, it is essential to thoroughly define normal aSyn function in neurons before pursuing aSyn reducing therapies. In this Research Topic the contributions of many aSyn experts describe original research, timely reviews, or perspectives regarding the role of aSyn to wellness and disease.

Surguchev and Surguchov review evidence that a normal function of all three synuclein homologs is a regulatory role in gene expression that occurs by their interactions with nucleic acids, transcription factors, and translation factors. Their main focus is on aSyn, as it has been more extensively studied. Emerging roles have been identified for aSyn in epigenetics by its binding interactions that affect DNA methylation, RNA-associated-gene-silencing, and histone acetylation. Further, genes involved in DNA repair are also modulated by aSyn, suggesting how the loss of soluble aSyn could impair cellular function. More evidence for important normal aSyn function is seen in well-controlled research from Benskey et al. who demonstrate a role for aSyn in maintaining nigrostriatal dopaminergic neuron viability. Using adult wild type rats in which they downregulate endogenous aSyn in substantia nigra with adeno-associated-virus short hairpin RNA, neuronal dysfunction is induced followed by neuronally-mediated inflammation and then nigral dopaminergic neuron loss. Control experiments in glutamatergic neurons of the rats have no loss of viability, confirming the key importance of aSyn in dopaminergic neurons. Added support comes from Vidal-Martinez et al. who review findings confirming that aSyn inhibits dopamine synthesis in neuronal cells (Perez et al., 2002), and inhibits insulin secretion from pancreatic ß-cells by aSyn binding the Kir6.2 subunit of K-ATP channels on insulin granules (Geng et al., 2011). Moreover, Kir6.2 has been shown to inhibit brain dopamine secretion (Avshalumov and Rice, 2003), suggesting that therapies that sustain aSyn:Kir6.2 interactions and enhance brain derived neurotrophic factor (BDNF) expression while blocking inflammation (Vargas-Medrano et al., 2014), may protect against diabetes and PD.

Mitochondrial impairment, which is common in PD, influences energy metabolism, homeostasis, the stress response, and apoptosis (Winklhofer and Haass, 2010). Vicario et al. review data showing that aSyn directly influences mitochondria by modulating their membrane potential, calcium homeostasis, cytochrome c release, ATP production, and fusion/fission. aSyn localization on mitochondria was first demonstrated in dopaminergic cells (Perez et al., 2002), and more recently using aSyn overexpressing dopaminergic cells others show that aSyn inhibits fusion and stimulates fission of mitochondria (Kamp et al., 2010). In that same study increasing wild type PINK1 or Parkin expression in dopaminergic cells reversed their mitochondrial deficits. Research from Creed and Goldberg in PINK1 knockout (−/−) rats demonstrates age-onset aSyn accumulation in synaptic vesicle pools, as well as spontaneous accumulation of insoluble aSyn in cortex, thalamus, striatum, and ventral midbrain. Curiously, aSyn pathology arises in the rats even though aSyn is not overexpressed. The authors propose that PARK1 −/− rats nicely model sporadic PD making them useful for assessing treatments aimed at slowing PD progression. Research by Cuvelier et al. reveal metabolic changes such as reduced body mass and less adiposity in aging male Thy-1 transgenic mice that express wild type human aSyn (Thy1-aSYN mice) on a C57BL/6-DBA/2 background (Rockenstein et al., 2002; Fleming et al., 2004). Thy1-aSYN males also exhibit increased spontaneous activity, lower food intake, and reduced energy expenditure than control mice. As metabolism and mitochondria are strongly linked, it could be instructive to assess the activity of mitochondria in adipose tissue from male Thy1-aSYN mice, especially in light of data in humans with persistent low body weights having higher mitochondrial activity in white adipose tissue (Ling et al., 2019).

Further regarding aSyn in neurons, Post et al. review interactions between aSyn, dopamine and calcium that underlie the loss of substantia nigra and locus coeruleus (LC) neurons in PD. In their multi-hit model they describe synergistic interactions between aSyn, calcium ions and dopamine that cause abnormal protein turnover that leads to nigral and LC vulnerability. The review by Betzer and Jensen reconsiders the calcium hypothesis which states that elevated intracellular neuronal calcium causes their demise. They present more recent findings showing that neurons undergoing a gradual build-up of aSyn, cytosolic calcium is actually reduced as aggregated aSyn binds to the sarcoplasmic-endoplasmic reticulum (ER) calcium ATPase (SERCA). The aggregated aSyn binding activates SERCA which reduces cytosolic calcium and overloads the ER with calcium (Betzer et al., 2018). They suggest that targeting abnormally activated SERCA or preventing the aSyn:SERCA interaction could yield therapies to slow PD. Butkovich et al. describe research on how a loss of LC norepinephrine (NE) and subsequent NE-effects reduce neurotrophic factor signaling, worsen central and peripheral inflammation, and alter innate and adaptive immune responses to accelerate PD progression. They note that in NE-producing cells and related animal models that overexpress aSyn, aSyn translocates to the nucleus where it interferes with transcription of dopamine ß-hydroxylase (DBH), the final enzyme in NE biosynthesis to reduce NE production (Kim et al., 2014). Loss of NE in noradrenergic neurons could be exacerbated by the upstream inhibition of tyrosine hydroxylase (Perez et al., 2002) and amino acid decarboxylase (Tehranian et al., 2006) by aSyn, producing lower levels of dopamine to convert to NE by DBH.

A final series of articles describes tools for elucidating aSyn structure, *in vivo* modeling of sporadic PD, and aSyn interactions with vesicle proteins. Dettmer reviews the use of rationally-designed aSyn variants to define properties of aSyn that are relevant to health and disease. Describing biochemical and cell biological aSyn data from various labs, he emphasizes how intact-cell approaches show how small changes in aSyn structure can contribute to PD and other synucleinopathies. In a perspectives article, Duffy et al. support the use of preformed fibrils of aSyn to model idiopathic PD to allow exploring relationships between aSyn aggregation and cellular toxicity. Preformed aSyn fibrils can be injected intrastriatally at endogenous levels after which over time they induce LB-like inclusions, neuroinflammation, and progressive nigrostriatal degeneration. Almandoz-Gil et al. show co-localization of aSyn with SNARE proteins in primary cortical neurons by *in situ* proximity ligation assays (PLA). PLA was previously used to corroborate aSyn interactions with other proteins or identify aSyn oligomers in PD brain. In beautiful images they show aSyn proximity with VAMP-2, SNAP-25, and syntaxin-1 in cell bodies and neurites, remarkably no differences were seen in neuronal aSyn:SNARE interactions from A30P aSyn transgenic and non-transgenic mice.

In conclusion, the papers presented in this Research Topic emphasize the importance of aSyn function that when exaggerated or disrupted may impair function and viability of neurons, pancreatic ß-cells, and other cells that utilize normal aSyn function. The data further suggest that therapies that help sustain normal levels of soluble aSyn could be highly protective.

## Author Contributions

RP authored the editorial by making direct intellectual contributions regarding the Research Topic.

### Conflict of Interest

RP is now the CEO of R&R Perez, LLC and declares that this editorial and any research described was done in the absence of any commercial or financial relationships that could be construed as a potential conflict of interest.
